# A Case of Concussion of the Brain, Which Terminated Fatally

**Published:** 1799-12

**Authors:** Charles Brown

**Affiliations:** Member *of the* Corporation *of* Surgeons, London


### *A Cafe of Concuffion of the Brain, which terminated fatally. By* Mr. Charles Brown, Member *of the* Corporation *of* Surgeons, London.

LAST Tuefday evening, about fix o'clock, I was fent for to a young gentleman in Guilford Street; who, by a fall from his horfe, on the pavement in Finfbury Square, had received a violent blow upon the left parietal bone. When I arrived at his houfe, which was about two hours after the accident, (being then but juft removed home) I found him lying in bed, vomiting every two minutes, and in a ftate of ftupor. His extremities were cold, the pupils very much contracted, and he had a frown on his face, as if he knew his own complaint. Upon examining the head, I found the integuments much bruifed; but the pericranium was not detached from the fkull. I immediately took away fixteen ounces of blood from the temporal artery on the affected fide; directed the head to be fhaved, and applied a large blifter all over it. The following mixture was prefcribed; of which, I ordered three table fpoonfuls to be taken every two hours, till it operated.

℞ Infus. fennæ ℥vij, tinc$^{t.}$ jalapii ʒjij, fyr. e fpin. cervin. ℥ifs, kali tartar ʒiij, tinc$^{t.}$ lavend. c. ℥fs. M. f$^{t.}$ mixt. purg.

The next morning I found him more fenfible; his pulfe was ftill full and hard, and his breathing was laborious. He took the whole of the mixture, before it operated; he then had two ftools: I repeated the mixture, and took away twelve ounces of blood from the median vein in his right arm: the blifter was cut and dreffed with the ung$^{t.}$ epi-fpaft. Before the evening, he had feveral ftools, after which he was feized with fhivering fits, and talked incoherently; I vifited him at ele-ven o'clock this evening, and directed pul. ipecac. comp. gr. xv to be taken in a draught, with twenty drops of vini antim. tart. every fix hours. The next day, I perceived no alteration; his pulfe ftill hard and full; his medicines had fweated him profufely, and his extremities felt warm. He paffed the whole of this day very reftlefs, and died about ten o'clock at night, juft as I was holding a confultation on his cafe with another furgeon. Having obtained permiffion, I opened his head next morning, and found both the veffels of the dura and pia ma-ter very turgid with blood. A layer of coagulable lymph adhered upon upon the inner surface of the dura mater, like an adventitious membrane. Suppuration had taken place, and a considerable quantity of pus lay between the membranes of the brain; the spinus or middle artery of the dura mater was uncommonly large in this subject.

*Hatton Garden, Nov.* 5, 1799. CHARLES BROWN.

